# Diastolic dysfunction assessed by cardiac magnetic resonance imaging tissue tracking on normal-thickness wall segments in hypertrophic cardiomyopathy

**DOI:** 10.1186/s12880-022-00955-7

**Published:** 2023-01-10

**Authors:** Jinhan Qiao, Peijun Zhao, Jianyao Lu, Lu Huang, Xiaoling Ma, Xiaoyue Zhou, Liming Xia

**Affiliations:** 1 Department of Radiology, Tongji Hospital, Tongji Medical College, Huazhong University of Science and Technology, No 1095 Jiefang Avenue, Qiaokou District, Wuhan, 430030 People’s Republic of China; 2 MR Collaboration, Siemens Healthcare Ltd., Shanghai, People’s Republic of China

**Keywords:** Hypertrophic cardiomyopathy, Strain, Diastolic function, Magnetic resonance imaging

## Abstract

**Objectives:**

Myocardial strain is reported to be a sensitive indicator of myocardial mechanical changes in patients with hypertrophic cardiomyopathy (HCM). The changes in the mechanics of the myocardium of normal wall thickness (< 12 mm) have yet to be well studied. This study aimed to evaluate the function of myocardial segments of normal thickness in patients with HCM.

**Methods:**

Sixty-three patients with HCM and 30 controls were retrospectively enrolled in this retrospective study. Cine imaging, native and post-contrast T1 maps, T2 maps, and late gadolinium enhancement were performed. In addition, regional myocardial strain was assessed by cardiac magnetic resonance-tissue tracking. Strain parameters were compared between the controls and HCM patients with segments of the myocardium of normal thickness. Subgroup analysis was conducted in obstructive and non-obstructive HCM. Lastly, *p* < 0.05 was considered statistically significant.

**Results:**

In normal-thickness myocardial segments of HCM (n = 716), diastolic peak strain rates (PSRs) were significantly lower than in the control group (n = 480) (radial, − 2.43 [− 3.36, − 1.78] vs. − 2.67 [− 3.58, − 1.96], *p* = 0.002; circumferential, 1.28 [1.01,1.60] vs. 1.39 [1.14, 1.78], *p* < 0.001; and longitudinal, 1.16 [0.75,1.51] vs. 1.28 [0.90, 1.71], *p* < 0.001). The normal-thickness segments showed no significant difference in systolic PSRs between HCM and the controls. In the subgroup analysis, significantly decreased diastolic PSRs were noted in both obstructive and non-obstructive HCM, compared with the controls (*p* < 0.05).

**Conclusions:**

Diastolic changes in myocardial mechanics were observed in normal-thickness segments of HCM, occurring before morphological remodeling and systolic dysfunction developed. This finding contributed to a better understanding of the mechanical pathophysiology of HCM with preserved left ventricular ejection fraction. It may potentially aid in predicting disease progression and risk stratification.

**Supplementary Information:**

The online version contains supplementary material available at 10.1186/s12880-022-00955-7.

## Background

Hypertrophic cardiomyopathy (HCM) is characterized by left ventricular (LV) thickening that cannot be explained by an abnormal load or other cardiac or systemic diseases [1, 2]. The incidence of HCM is 1:500–1:200 [3, 4]. Notably, the annual incidence of cardiovascular death among adult patients with HCM is 1–2%, primarily from sudden cardiac death and heart failure [1].

The end-stage of HCM is characterized by systolic dysfunction (left ventricular ejection fraction [LVEF] < 50%) [5]. Among these patients, the risk of death was tenfold higher than in those with normal systolic dysfunction [5, 6]. In the early stages of HCM, LVEF tends to be preserved or pathologically elevated [4, 7]. In contrast, the strain parameters at this stage are impaired. They are derived from cardiac magnetic resonance tissue tracking (CMR-TT) or echocardiography speckle tracking imaging, which detect regional and global myocardial dysfunction [8, 9]. Therefore, the strain was believed to be more sensitive in detecting early changes in myocardial mechanics than LVEF in HCM patients [10, 11]. In addition, the strain derived from CMR-TT is more reproducible than from echocardiography [12].

In HCM patients with normal LVEF, the non-hypertrophied segments motion has been shown to be impaired or display compensatory hypercontractility [8, 9, 13]. Notably, segments with a maximal LV wall thickness < 15 mm were classified as non-hypertrophied in patients with HCM [8, 14]. In contrast, segments in healthy controls with a maximal LV wall thickness < 12 mm were defined as non-hypertrophied [15]. In HCM patients, the dysfunction of segments with a thickness of 12–15 mm is easy to understand since the structural hypertrophy has occurred. However, it is unknown whether mechanical myocardial changes occur in wall segments of normal thickness.

This study aimed to investigate the regional function of segments with normal thickness using CMR-TT, to better understand the pathophysiology of HCM with preserved LVEF. In addition, the goal is to find a new biomarker in HCM patients that is potentially useful for monitoring the disease progression.

## Methods

### Study population

**Fig. 1:**
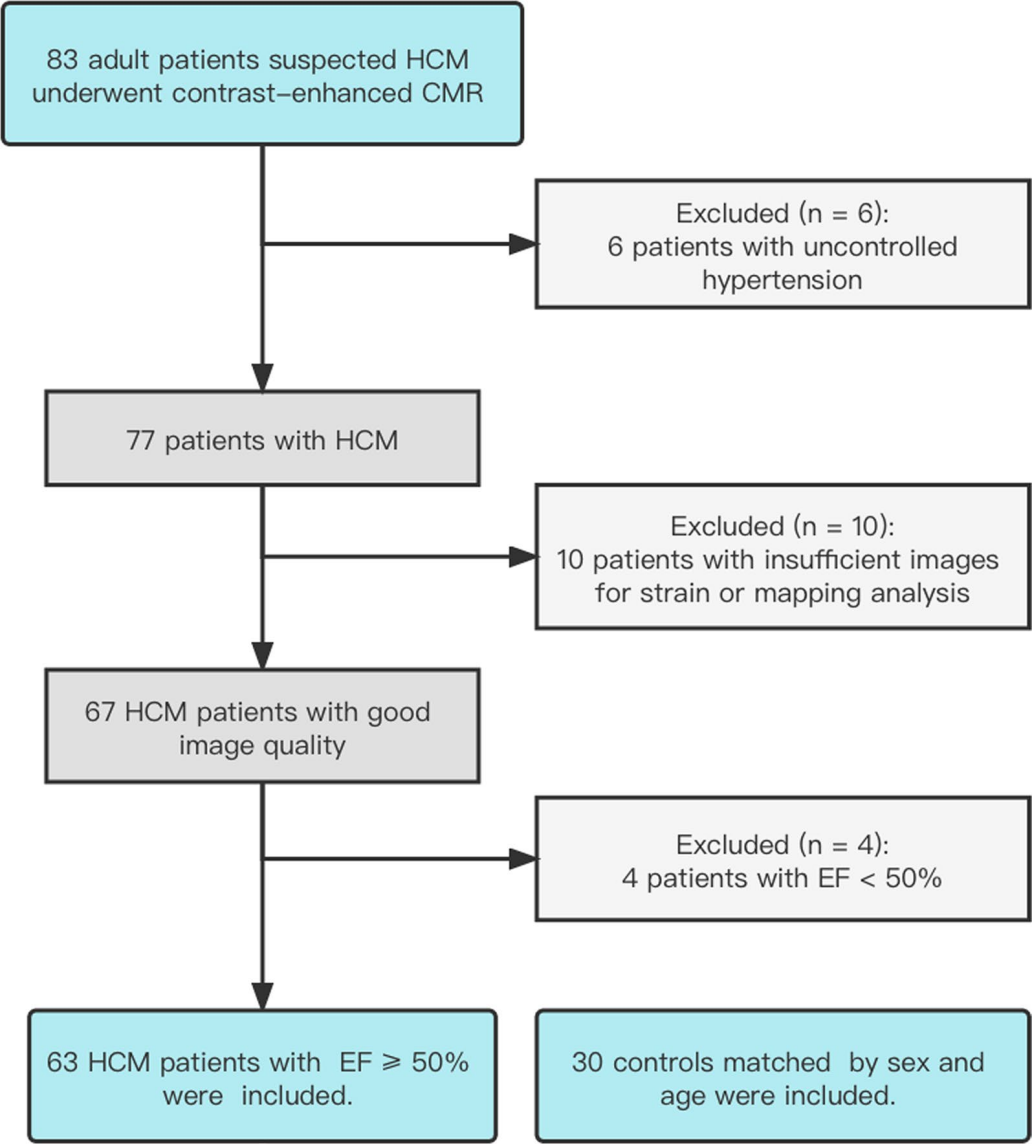
Study flow diagram. CMR, cardiac magnetic resonance; HCM, hypertrophic cardiomyopathy; EF, ejection fraction

We retrospectively enrolled 63 HCM patients who underwent contrast-enhanced CMR imaging in our institution from May 2018 to January 2020 and 30 healthy controls matched for age and sex (Fig. 1). The control group showed no abnormalities on contrast-enhanced CMR. The HCM inclusion criteria were: (1) maximum LV wall thickness ≥ 15 mm, or ≥ 13 mm with family history, and (2) age ≥ 18 years old. The exclusion criteria were: (1) the presence of other conditions that could lead to LV hypertrophy, (2) image quality too poor for segmental analysis, and (3) LVEF < 50%. The clinical history, serum markers, and echocardiographic data were collected for all subjects. This study was approved by the Institutional Ethics Committee, and written informed consent was waived due to the retrospective design.

### Image acquisition

All subjects underwent a CMR scan with an 18-channel phased-array body coil on a 3 T scanner (MAGNETOM Skyra, Siemens Healthcare, Erlangen, Germany). CMR protocols included cine in the short- and long-axis (2-, 3-, and 4-chamber), native and post-contrast T1 mapping, T2 mapping in three short axis slices (basal, middle, and apical), and late gadolinium enhancement (LGE) images at the exact location as the short-axis cine slices.

A standard breath-hold cine imaging with steady-state free precession (SSFP) sequence was acquired. The parameters were as follows: echo time (TE) = 1.4 ms; repetition time (TR) = 3.1 ms; flip angle (FA) = 55°; slice thickness = 8 mm; voxel size = 1.9 × 1.9 mm^2^; slice gap = 2 mm; bandwidth = 965 Hz/pixel; FOV = 360 × 360 mm^2^; and 25 calculated phases per heartbeat.

T1 mapping was acquired by using a single breath-hold Modified Look-Locker Inversion recovery sequence. In addition, 5b(3b)3b and 4b(1b)3b(1b)2b schemes were used for the native and post-contrast T1 imaging, respectively, 15–20 min after administration of a contrast agent dose (0.2 mL/kg of MultiHance, Bracco Diagnostics). The T1 parameters were as follows: TE = 1.2 ms; TR = 3.8 ms; FA = 35°; slice thickness = 5 mm; and voxel size = 1.4 × 1.4 mm^2^. Furthermore, T2 mapping was generated using a T2-prepared bSSFP sequence with three T2 preparation times: 0, 24, and 55 ms. The T2 parameters were as follows: TE = 1.41 ms; TR = 3.3 ms; FA = 12°; slice thickness = 5 mm; voxel size = 1.9 × 1.9 mm^2^; and acquisition matrix = 206 × 256.

LGE imaging was performed 10–15 min after administration of the contrast agent using an inversion recovery prepared pulse and segmented FLASH sequence with a phase-sensitive reconstruction technique. Imaging parameters were as follows: TE = 1.2 ms; TR = 5.2 ms; FA = 55°; slice thickness = 8.0 mm; and voxel size = 1.5 × 1.5 mm^2^.

### Image analysis

All CMR images were analyzed with commercial software (CVI 42, version 5.11, Circle Cardiovascular Imaging, Calgary, Canada) according to the 16-segment American Heart Association (AHA) model [8].

**Fig. 2:**
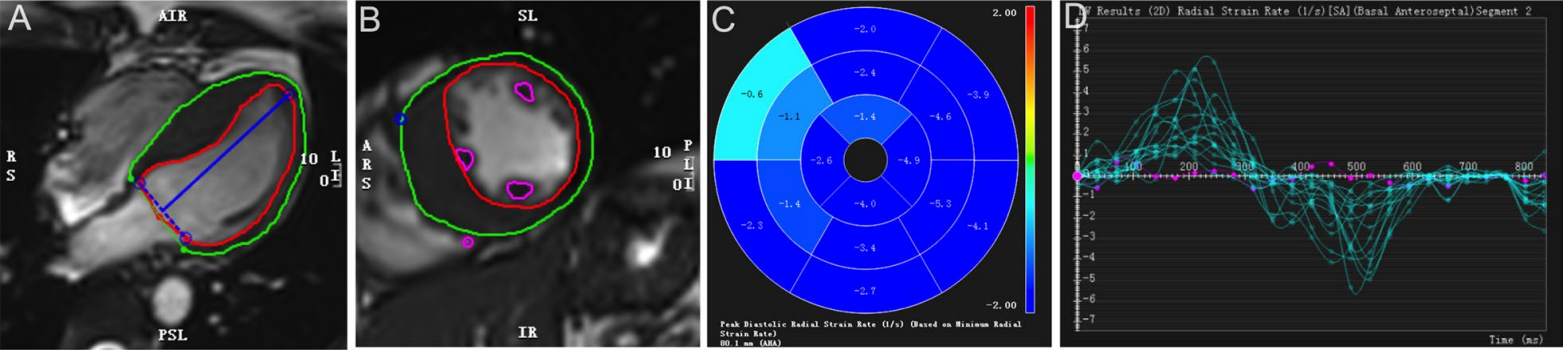
CMR Tissue Tracking contours. The endo- (red curve) and epi-cardial (green curve) borders on the cine images of the four-chamber (**A**) and short axis (**B**). The peak circumferential diastolic strain rate results are shown in polarmap (**C**) and diagram (**D**)

Contours in the endo- and epi-myocardium at the LV end-diastolic phase without papillary muscle were automatically sketched and manually corrected on a short-axis cine by a radiologist with 3 years of experience using the short-3D module (Figure 2). In addition, the LV functional parameters were obtained automatically.

Then, the cine images of the short and long axes were loaded into the Tissue Tracking module to outline the endocardium and epicardium and generate segmental strain parameters, including the peak strain (PS), systolic peak strain rate (PSR) and diastolic PSR in the radial, circumferential, and longitudinal directions.

According to the maximum thickness of the myocardium, the total HCM segments were divided into group A (< 12mm) [15], group B (12–15 mm), and group C (> 15mm) [8, 14]. We further compared the strain within the subgroups of obstructive and non-obstructive HCM to exclude the influence of an obstructive left ventricular outflow tract (LVOT) on this strain parameter. Notably, a maximum LVOT gradient of ≥ 30 mmHg at rest or with provocation represented the presence of obstruction, based on the result of echocardiography [16].

Segmental native T1 and T2 values were obtained by manually delineating the endocardium and epicardium of the entire LV myocardial region (which included the LGE-positive regions) on the T1 and T2 mapping images [17]. Then, the extracellular volume (ECV) was calculated using the hematocrit (Hct) and the myocardial and blood intensity before and after enhancement: ECV = (1/T1_*myo post* _*− *1/T1_*myo pre*_) × (1 *− *Hct)/(1/T1_*blood post* _*− *1/T1_*blood pre*_)^16^. T1_*myo post*_ and T1_*myo pre*_ represented the post- and pre-contrast myocardial T1 values, respectively, whereas T1_*blood post*_ and T1 _*blood pre*_ were the post- and pre-contrast blood T1 value [18]. The Hct used was from the routine blood test conducted on the day of CMR scanning. Positive LGE lesions were defined as a signal intensity greater than six standard deviations above the mean signal intensity of the remote reference myocardium [19, 20]. In addition, the LGE ratios were calculated according to the LGE volume and segmental LV myocardial volume.

We randomly selected 20 patients for inter- and intra-observer agreement analysis. The strain parameters were measured independently by two blinded observers (J.H.Q. and P.J.Z, with 3 and 5 years of CMR diagnostic experience, respectively), one of whom re-measured the strains after one month (J.H.Q).

### Statistical analysis

All statistical analyses were performed using SPSS Statistics 22 (IBM, Chicago, USA). Categorical variables were presented as frequencies (percentages) and analyzed with Pearson’s chi-squared test. The normality distribution of the continuous data was determined by the Shapiro–Wilk test. Continuous variables were expressed as mean ± standard deviation or median (interquartile range). An independent *t*-test or Mann–Whitney *U*-test was performed to compare two groups of continuous variables. The Kruskal–Wallis rank test was used to analyze the differences among three or more groups. In addition, Bonferroni’s correction was conducted to perform post-hoc comparisons. The association between myocardial thickness and other quantitative myocardial parameters (e.g., strain indexes and tissue characteristics) was evaluated with the Spearman correlation, which had the following values: Spearman correlation value of 0.00–0.10 was considered a negligible correlation; 0.10–0.39, weak correlation; 0.40–0.69, moderate correlation; 0.70–0.89, strong correlation; and 0.90–1.00, very strong correlation [21]. Furthermore, the intraclass correlation coefficient (ICC) was applied to assess the inter- and intra-observer reproducibility variability, in which a value > 0.75 represented good agreement. Lastly, a *p*-value < 0.05 was considered statistically significant.

## Results

Table 1 shows the clinical characteristics of the cases and controls. Twenty-one (33.3%) patients were diagnosed as having obstructive HCM. Among the HCM patients, 46 (73.0%) reported experiencing chest pain, and four (6.3%) had a history of syncope.

**Table 1:** Clinical characteristics in HCM patients and controls

Parameter	Controls (n = 30)	Patients (n = 63)	*p-*value
Age (year)	50 ± 11	54 ± 11	0.07
Sex, male (%)	18 (60.0)	43 (68.3)	0.06
Height (m)	1.65 ± 0.07	1.66 ± 0.07	0.42
Weight (kg)	67.7 ± 12.2	68.1 ± 12.1	0.87
BMI (kg/m^2^)	24.8 ± 4.3	24.4 ± 3.0	0.65
BSA (m^2^)	1.72 ± 0.18	1.73 ± 0.19	0.75
Heart rate (beats/min)	66 (60, 76)	68 (63, 74)	0.38
Systolic BP (mm Hg)	120 (112, 128)	125 (118, 135)	0.06
Diastolic BP (mm Hg)	80 (73, 82)	80 (71, 88)	0.61
Drinking (%)	4 (13.3)	7 (11.1)	0.79
Smoking (%)	5 (16.7)	17 (27.0)	0.56
Hct (%)	40.9 ± 4.7	40.4 ± 4.7	0.38

The values are presented as frequencies (percentages) or means ± standard deviation or medians (interquartile range)

*HCM*, hypertrophic cardiomyopathy; *BMI*, body mass index; *BSA*, body surface area; *BP*, blood pressure; *Hct*, hematocrit

### Differences in cardiac function

Compared with the controls, patients with HCM had a significantly increased myocardial mass index, cardiac output, and stroke volume (*p* < 0.001, *p* = 0.03, and *p* = 0.04, respectively) (Table 2). However, there were no statistically significant differences between the HCM patients and controls in LVEF, end-diastolic volume, or end-systolic volume (*p* = 0.07, *p* = 0.10, and *p* = 0.90, respectively).

**Table 2:** CMR imaging findings in HCM patients and controls

Parameter	Controls (n = 30)	Patients (n = 63)	*p-*value
EF (%)	62.5 ± 7.9	65.5 ± 6.8	0.07
CO (L)	4.1 (3.5, 5.8)	5.4 (4.7, 6.9)	**0.03**
EDV (mL)	111.0 ± 23.5	122.1 ± 32.5	0.10
ESV (mL)	39.2 (33.5, 50.6)	40.9 (32.9, 47.8)	0.90
SV (mL)	69.6 ± 18.7	80.3 ± 24.0	**0.04**
EDV/BSA (mL/m^2^)	62.9 ± 12.3	68.6 ± 15.3	0.08
ESV/BSA (mL/m^2^)	23.1 (18.7, 27.8)	23.1 (18.7, 27.7)	0.90
SV /BSA (mL/m^2^)	39.3 ± 9.7	45.0 ± 11.4	**0.02**
LV mass (g)	72.5 (64.8, 82.4)	121.4 (100.3, 138.9)	** < 0.001**
LV mass/BSA (g/m^2^)	41.8 (37.3, 44.8)	68.5 (59.0, 79.3)	** < 0.001**

The values are presented as mean ± standard deviation or median (interquartile range). Bold values indicate *p* < 0.05

*CMR*, cardiac magnetic resonance; *HCM*, hypertrophic cardiomyopathy; *EF*, ejection fraction; *CO*, cardiac output; *EDV*, end diastolic volume; *ESV*, end systolic volume; *SV*, stroke volume; *BSA*, body surface area; *LV*,left ventricle/ventricular

### Comparison of strain parameters and tissue characteristics

There were 716 segments in group A, 111 in group B, and 181 in group C. As shown in Table 3, the diastolic PSR values in group A were all lower than in the control group in the radial, circumferential, and longitudinal directions (all *p* < 0.05). Notably, there was a significant difference in the native T1 (*p* < 0.001) between group A and the control group but no such difference in T2 (*p* = 0. 59) or ECV (*p* = 0. 99).

**Table 3:** Strain parameters and tissue characteristics in different groups

	Control (n = 480)	Group A (n = 716)	Group B (n = 111)	Group C (n = 181)	*p-*value
*Strain parameters*					
Radial PS (%)	37.30 (29.00, 48.19)	36.64 (28.58,47.58)	29.04 (22.34, 36.86)^*#^	25.51 (18.02, 33.02)^*#^	** < 0.001**
Circumferential PS (%)	− 20.83 (− 23.80, − 17.71)	− 20.53 (− 23.73, − 17.56)	− 17.90 (− 20.66, − 15.04)^*#^	− 16.46 (− 19.06, − 12.69)^*#^	** < 0.001**
Longitudinal PS (%)	− 15.70 (− 20.78, − 10.62)	− 15.68 (− 20.56, − 9.64)	− 13.73 (− 17.23, − 8.66)^*#^	− 10.63 (− 14.83, − 6.58)^*#^	** < 0.001**
Radial systolic PSR (1/s)	2.06 (1.58, 2.81)	2.28 (1.69, 3.00)^*^	1.75 (1.37, 2.32)^*#^	1.51 (1.14, 2.03)^*#^	** < 0.001**
Circumferential systolic PSR (1/s)	− 1.18 (− 1.50, − 0.99)	− 1.28 (− 1.55, − 1.02)	− 1.14 (− 1.41, − 0.94) ^#^	− 1.02 (− 1.27, − 0.83)^*#$^	** < 0.001**
Longitudinal systolic PSR (1/s)	− 1.10 (− 1.49, − 0.76)	− 1.14 (− 1.53, − 0.71)	− 1.02 (− 1.42, − 0.63)	− 0.93 (− 1.23, − 0.61)^*#^	** < 0.001**
Radial diastolic PSR (1/s)	− 2.67 (− 3.58, − 1.96)	− 2.43 (− 3.36, − 1.78)^*^	− 1.71 (− 2.27, − 1.24)^*#^	− 1.38 (− 1.94, − 1.02)^*#^	** < 0.001**
Circumferential diastolic PSR (1/s)	1.39 (1.14, 1.78)	1.28 (1.01,1.60)^*^	1.00 (0.80, 1.20)^*#^	0.90 (0.73, 1.10)^*#^	** < 0.001**
Longitudinal diastolic PSR (1/s)	1.28 (0.90, 1.71)	1.16 (0.75,1.51)^*^	0.96 (0.67, 1.23)^*#^	0.88 (0.60, 1.21)^*#^	** < 0.001**
*Tissue characteristics*					
LGE, n (%)	0 (0)	30 (4.1)^*^	14 (12.6)^*#^	62 (34.2)^*#$^	** < 0.001**
Mean native T1 (ms)	1231.2 (1194.7, 1264.2)	1252.2 (1222.8, 1287.7)^*^	1278.7 (1244.8, 1305.2)^*#^	1293.1 (1268.7, 1332.4)^*#$^	** < 0.001**
Mean T2 (ms)	39.8 (37.9, 41.9)	39.8 (38.4, 41.4)	39.4 (38.1, 41.2)	39.9 (38.7, 41.3)	0.59
Mean ECV (%)	25.0 (22.9, 27.0)	25.2 (22.8, 27.7)	25.5 (23.3, 29.4)	26.6 (23.9, 29.7)^*#^	** < 0.001**

The groups (A, B, C) of HCM are divided according to the maximum thickness of the segmental myocardium: Group A, < 12 mm; Group B, 12-15 mm; Group C, > 15 mm. Analysis of the segments is based on the 16-segment AHA model

The values are presented as frequencies (percentages) or median (interquartile range). Bold values indicate *p* < 0.05 when compared among the four groups

*, vs. control, *p* < 0.05;

^#^, vs. Group A, *p* < 0.05;

^$^, vs. Group B, *p* < 0.05

*PS*, peak strain; *PSR*, peak strain rate; *LGE*, late gadolinium enhancement; *ECV*, extracellular volume; *HCM*, hypertrophic cardiomyopathy, *AHA*, American Heart Association

### Comparison of strain parameters among subgroups

**Fig. 3:**
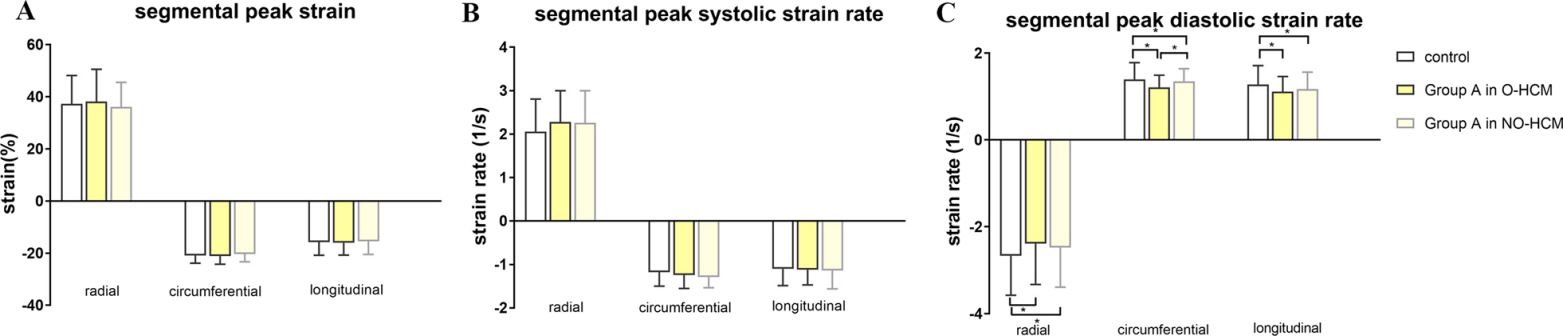
Subgroup analysis of the strain parameters in group A (< 12 mm), including segmental peak strain (**A**), segmental peak systolic strain rate (**B**), and segmental peak diastolic strain rate (**C**). The bars indicate the median values with the interquartile range. *p < 0.05. O-HCM, obstructive hypertrophic cardiomyopathy; NO-HCM, non-obstructive hypertrophic cardiomyopathy

Further analysis of segmental strain in the normal-thickness wall segments are shown in Fig. 3 for patients with obstructive HCM, non-obstructive HCM, and controls. There were decreased diastolic PSRs in both subgroups compared with the controls (both *p* < 0.05). In addition, the demographic characteristics of the subgroups are shown in the supplementary materials (Additional file 1: Table S1).

### Correlation analysis

As demonstrated in Table 4, the radial diastolic PSR was moderately associated with the end-diastolic wall thickness (EDTH) (*r* = 0.44, *p* < 0.001). The EDTH was weakly correlated with the extent of LGE (*r* = 0.35, *p* < 0.001) and native T1 (*r* = 0.34, *p* < 0.001).

**Table 4:** Bivariate analysis regarding the association of myocardial quantitative myocardial parameters and myocardial thickness

	EDTH	
	r	*p-*value
Radial diastolic PSR	**0.44**	** < 0.001**
Circumferential diastolic PSR	**−** **0.38**	** < 0.001**
Longitudinal diastolic PSR	**−** **0.16**	** < 0.001**
LGE ratio	**0.35**	** < 0.001**
Native T1	**0.34**	** < 0.001**
T2	− 0.06	0.064
ECV	0.05	0.132

Bold values indicate *p* < 0.05

*EDTH*, end-diastolic wall thickness; *PSR*, peak strain rate; *LGE*, late gadolinium enhancement; *ECV*, extracellular volume

### Inter- and intra-observer agreements

As shown in Table 5, all inter-observer ICCs were above 0.848 and the intra-observer ICCs were higher than 0.862, indicating good observer agreement and reliability.

**Table 5:** Inter- and intra-observer variability of strain by the ICC analysis

	Inter-observer		Intra-observer	
	ICC	95% CI	ICC	95% CI
Radial PS (%)	0.948	0.927–0.965	0.961	0.927–0.978
Circumferential PS (%)	0.963	0.920–0.975	0.967	0.945–0.981
Longitudinal PS (%)	0.935	0.907–0.954	0.955	0.934–0.964
Radial PSSR (1/s)	0.869	0.843–0.891	0.878	0.849–0.896
Circumferential PSSR (1/s)	0.883	0.853–0.914	0.898	0.866–0.911
Longitudinal PSSR (1/s)	0.848	0.835–0.876	0.862	0.843–0.886
Radial PDSR (1/s)	0.891	0.875–0.914	0.889	0.843–0.902
Circumferential PDSR (1/s)	0.894	0.866–0.917	0.906	0.858–0.916
Longitudinal PDSR(1/s)	0.859	0.846–0.887	0.881	0.867–0.896

*ICC*, intraclass correlation coefficient, *CI*, confidence interval, *PS*, peak strain; *PDSR*, peak diastolic strain rate; *PSSR*, peak systolic strain rate

## Discussion

We found that in HCM patients, the diastolic PSR of segments with normal thickness (< 12 mm) were impaired in the radial, circumferential, and longitudinal directions compared with the controls. This finding suggested that functional remodeling with changes in diastolic dysfunction may precede morphological remodeling in normal-thickness HCM segments.

We observed that diastolic PSR decreased in all segments. In contrast, PS and systolic PSR were impaired in segments with a thickness ≥ 12 mm [8], indicating that diastolic function was impaired prior to morphological remodeling and systolic dysfunction. Notably, diastolic dysfunction prior to systolic impairment has been described in HCM patients [9, 22]. In addition, functional remodeling in non-hypertrophic segments has also been reported in a previous study [8]. Our study showed that mechanical impairment in HCM involved the entire LV myocardium (including segments with normal-thickness), not only the hypertrophic myocardium. The finding of impaired diastolic function in normal-thickness wall segments contributed to a better understanding of the pathophysiology of HCM, which may help predict disease progression. The diastolic dysfunction of HCM may be associated with changes in the conformation of myosin and molecular effects such as increased calcium ion sensitivity [23, 24]. Interestingly, diastolic abnormalities were confirmed in preclinical sarcomere mutation carriers, whose segments were of normal thickness [25, 26].

In the subgroup analysis, there were no significant differences between obstructive and non-obstructive HCM in diastolic PSR for the normal-thickness wall segments. However, Zhao et al. [27] reported differences in global strain between obstructive and non-obstructive HCM. This finding may be explained by the fact that we focused on segmental strain while Zhao et al. focused on the global strain. Furthermore, obstructive HCM was a global consequence of the pressure gradient. Due to the different ranges of LV hypertrophy, global and segmental strains may vary in HCM. However, there are few reports on segmental strain in obstructive HCM, so this difference might be difficult to confirm. She et al. [16] found that free wall strain was not significantly different between obstructive and non-obstructive HCM. More studies on segmental strain parameters are needed to explore the differences between obstructive and non-obstructive HCM.

Consistent with the study by Swoboda et al. [14], this study found the increased native T1 and the preserved ECV in the normal-thickness wall segments, which indicated that native T1 might be more sensitive to changes than ECV in HCM. Similarly, Puntmann et al. [28] observed that native T1 provided a higher diagnostic accuracy than ECV for the discrimination of healthy controls and HCM. Moreover, Hinojar et al. [29] demonstrated that native T1 increased prior to morphological changes in individuals who were identified carriers of the relevant sarcomere–gene mutations for HCM but had no evidence of LV hypertrophy. Therefore, native T1 might be a potential marker for monitoring HCM progression or the response to clinical intervention [14].

We performed a correlation analysis to explore myocardial changes that occurred with the variation in wall thickness. The segmental strain index showed a better correlation with myocardial thickness than LGE, consistent with previous reports [8, 30]. This finding suggested that HCM progression could be monitored by the strain index, particularly in the radial direction. Moreover, the higher correlation of EDTH with the radial direction than the other two directions may be related to the fact that at an early stage, myocardial hypertrophy induced increased wall thickness in the radial direction [8]. There is a weak correlation between LGE and native T1with EDTH, consistent with previous studies  [30, 31]. Notably, the presence of LGE increases the stiffness of the ventricular wall, reportedly affecting patients’ prognosis [32].

Our study had several limitations. First, there was a lack of genetic results for our participants, and the effects of different genotypes on normal-thickness segment strain should be explored in future studies. Second, this study only included patients with localized basal septal hypertrophy and reverse curvature septal hypertrophy categories as reported in a previous study [33], due to the limited sample size. Patients with apical HCM should be investigated in future studies. Third, the study sample size was relatively small. More patients should be included in future studies. Finally, we did not compare the strain in CMR and the strain in echocardiography speckle tracking imaging.

## Conclusion

In conclusion, ventricular diastolic dysfunction was observed in normal-thickness wall segments in HCM patients, a process that preceded morphological remodeling. This finding contributed to a better understanding of HCM pathophysiology of and may potentially help predict disease progression and risk stratification in patients with HCM.

## Supplementary Information

**Additional file 1**: **Table S1**. Clinical characteristics and CMR imaging findings in obstructive and non-obstructive HCM patients and controls

## Data Availability

The data that support the findings of this study are available on request from the corresponding author.

## Abbreviations

HCM: Hypertrophic cardiomyopathy
LGE: Late gadolinium enhancement
CMR-TT: Cardiac magnetic resonance tissue tracking
PS: Peak strain
PSR: Peak strain rate
EDTH: End-diastolic thickness
LVEF: Left ventricular ejection fraction
LV: Left ventricular
TE: Echo time
TR: Repetition time
FA: Flip angle
AHA: American heart association
LVOT: Left ventricular outflow tract
ECV: Extracellular volume
ICC: Intraclass correlation coefficient
