# Supplementary material for: Diastolic dysfunction assessed by cardiac magnetic resonance imaging tissue tracking on normal-thickness wall segments in hypertrophic cardiomyopathy
Source: BMC Med Imaging. 2023 Jan 10;23:7. doi: 10.1186/s12880-022-00955-7 (PMC9830799; doi:10.1186/s12880-022-00955-7)
Supplement: Supplementary file 1 — Additional file 1: Table S1. Clinical characteristics and CMR imaging findings in obstructive and non-obstructive HCM patients and controls [file 12880_2022_955_MOESM1_ESM.docx]

**Supplementary Table. Clinical characteristics and CMR imaging findings in obstructive and non-obstructive HCM patients and controls.**

| **Parameter** | **Controls (n = 30)** | **Non-obstructive HCM (n = 42)** | **Obstructive HCM (n = 21)** | ***P* value** |
| --- | --- | --- | --- | --- |
| Age (year) | 50 ± 11 | 54 ± 12 | 55 ± 11 | 0.29 |
| Gender male (%) | 18 (60.0) | 29 (69.0) | 14 (66.7) | 0.72 |
| Height (m) | 1.65 ± 0.07 | 1.67 ± 0.07 | 1.66 ± 0.08 | 0.68 |
| Weight (kg) | 67.7 ± 12.2 | 69.0 ± 12.7 | 66.5 ± 10.9 | 0.73 |
| BMI (kg/m^2^） | 24.8 ± 4.3 | 24.6 ± 3.1 | 24.0 ± 2.8 | 0.72 |
| BSA (m^2^) | 1.72 ± 0.18 | 1.75 ± 0.20 | 1.71 ± 0.18 | 0.73 |
| Heart rate (beats/min) | 66 (60, 76) | 69 (62, 76) | 67 (64, 69) | 0.42 |
| Systolic BP (mm Hg) | 120 (112, 128) | 127 (120, 137) | 122 (109, 135) | 0.06 |
| Diastolic BP (mm Hg) | 80 (73, 82) | 80 (72, 93) | 77 (70, 84) | 0.20 |
| Drinking (%) | 4 (13.3) | 3 (7.1) | 4 (19.0) | 0.16 |
| Smoking (%) | 5 (16.7) | 12 (28.6) | 5 (23.8) | 0.77 |
| Chest pain (%) | 0 (0) | 28 (66.7) | 18 (85.7) | 0.11 |
| Syncope | 0 (0) | 1 (2.3) | 3 (14.3) | 0.07 |
| Hct (%) | 40.9 ± 4.7 | 41.1 ± 4.4 | 38.9 ± 5.0 | 0.14 |
| EF (%) | 62.5 ± 7.9 | 64.1 ± 6.6 | 68.3 ± 6.4 ^*^ | **0.01** |
| CO (L) | 4.1 (3.5, 5.8) | 5.4 (4.6, 6.8) | 5.4 (4.5, 7.4) | 0.07 |
| EDV (mL) | 111.0 ± 23.5 | 121.0 ± 33.1 | 124.2 ± 31.8 | 0.24 |
| ESV (mL) | 41.4 ± 11.7 | 43.4 ± 14.1 | 38.6 ± 9.6 | 0.35 |
| SV (mL) | 64.9 (59.3, 76.4) | 76.0 (61.9, 88.0) | 83.8 (62.8, 106.7) ^*^ | **0.04** |
| EDV/BSA (mL/m^2^) | 62.9 ± 12.3 | 67.4 ± 14.7 | 70.9 ± 16.5 | 0.14 |
| ESV/BSA (mL/m^2^) | 23.6 ± 6.7 | 24.2 ± 7.0 | 22.3 ± 6.1 | 0.55 |
| SV /BSA (mL/m^2^) | 39.3 ± 9.7 | 43.2 ± 10.2 | 48.6 ± 12.8 ^*^ | **0.01** |
| LV mass (g) | 73.9 ± 13.7 | 120.5 ± 31.2^*^ | 137.6 ± 39.9 ^*^ | **<0.001** |
| LV mass/BSA (g/m^2^) | 41.8 (37.3, 44.8) | 66.1 (54.7, 78.0) ^*^ | 75.2 (65.6, 87.7) ^*^ | **<0.001** |

The values are presented as frequency (percentages) or mean ± standard deviation or median (interquartile range). Bold values indicate p < 0.05 when compared among the three groups. ^*^, vs. control, p < 0.05.

CMR = cardiac magnetic resonance; HCM = hypertrophic cardiomyopathy; BMI = body mass index; BSA = body surface area; BP = blood pressure; Hct = hematocrit; EF = ejection fraction; CO = cardiac output; EDV = end diastolic volume; ESV = end systolic volume; SV = stroke volume; LV = left ventricular.
